# Supplementary material for: An ensemble penalized regression method for multi-ancestry polygenic risk prediction
Source: Nat Commun. 2024 Apr 15;15:3238. doi: 10.1038/s41467-024-47357-7 (PMC11271575; doi:10.1038/s41467-024-47357-7)
Supplement: Supplementary file 3 — Description of Additional Supplementary Files [file 41467_2024_47357_MOESM3_ESM.pdf]

Description of Additional Supplementary Files

File Name: Supplementary Data 1 (SD1)
Description: The complete result table (R^2) for simulation analysis under the assumed genetic architecture of strong negative selection (fixed common-SNP heritability).

File Name: Supplementary Data 2 (SD2)
Description: The complete result table (R^2) for simulation analysis under the assumed genetic architecture of mild negative selection (fixed common-SNP heritability).

File Name: Supplementary Data 3 (SD3)
Description: The complete result table (R^2) for simulation analysis under the assumed genetic architecture of no negative selection (fixed common-SNP heritability).

File Name: Supplementary Data 4 (SD4)
Description: The complete result table (R^2) for simulation analysis under the assumed genetic architecture of strong negative selection (fixed per-SNP heritability).

File Name: Supplementary Data 5 (SD5)
Description: The complete result table (R^2) for simulation analysis under the assumed genetic architecture of strong negative selection (fixed per-SNP heritability and less genetic correlation across populations).

File Name: Supplementary Data 6 (SD6)
Description: Computation time and memory usage for PROSPER and PRS-CSx.

File Name: Supplementary Data 7 (SD7)
Description: The detailed info of real GWAS data from 23andMe, GLGC, and AoU. Info includes: names of traits, GWAS ancestry, GWAS sample sizes, and number of SNPs.

File Name: Supplementary Data 8 (SD8)
Description: The detailed info of real individual-level data from 23andMe and UKBB. Info includes: names of traits, GWAS ancestry, tuning sample sizes, validation sample sizes, and data analyses that using this source of individual data.

File Name: Supplementary Data 9 (SD9)
Description: The complete result table (R^2or AUC) for analysis of traits and diseases in 23andMe.

File Name: Supplementary Data 10 (SD10)
Description: The percentage of improvement of PROSPER over other alternative methods for traits and diseases in 23andMe.

File Name: Supplementary Data 11 (SD11)
Description: The complete result table (R^2) for analysis of traits in GLGC.

File Name: Supplementary Data 12 (SD12)
Description: The percentage of improvement of PROSPER over other alternative methods for traits in GLGC.

File Name: Supplementary Data 13 (SD13)
Description: The complete result table (R^2) for analysis of traits in AoU.

File Name: Supplementary Data 14 (SD14)
Description: The percentage of improvement of PROSPER over other alternative methods for traits in AoU.

File Name: Supplementary Data 15 (SD15)
Description: The comparison of PROSPER and advanced weighted lassosum2 (with super learning across all tuning parameters and ancestries) in simulations.

File Name: Supplementary Data 16 (SD16)
Description: The comparison of PROSPER and advanced weighted lassosum2 (with super learning across all tuning parameters and ancestries) in real data.

File Name: Supplementary Data 17 (SD17)
Description: The sensitivity analysis allowing more flexible tuning parameters to accommodate larger genetic distance of the African (AFR) population from the others

File Name: Source_Data.zip
Description: Source data of Supplementary Figure 1 (Optimal tuning parameter lambda in lasso) and Supplementary Figure 13 (The relationship between tuning sample size and predictive R^2).
