# Supplementary material for: An ensemble penalized regression method for multi-ancestry polygenic risk prediction
Source: Nat Commun. 2024 Apr 15;15:3238. doi: 10.1038/s41467-024-47357-7 (PMC11271575; doi:10.1038/s41467-024-47357-7)
Supplement: Supplementary file 5 — Reporting Summary [file 41467_2024_47357_MOESM5_ESM.pdf]

nature portfolio

Corresponding author(s): Jingning Zhang and Nilanjan Chatterjee

Last updated by author(s): Mar 26, 2024

Reporting Summary

Nature Portfolio wishes to improve the reproducibility of the work that we publish. This form provides structure for consistency and transparency in reporting. For further information on Nature Portfolio policies, see our Editorial Policies and the Editorial Policy Checklist.

Statistics

For all statistical analyses, confirm that the following items are present in the figure legend, table legend, main text, or Methods section.

n/a	Confirmed	
☐	☒	The exact sample size (*n*) for each experimental group/condition, given as a discrete number and unit of measurement
☐	☒	A statement on whether measurements were taken from distinct samples or whether the same sample was measured repeatedly
☒	☐	The statistical test(s) used AND whether they are one- or two-sided<br>*Only common tests should be described solely by name; describe more complex techniques in the Methods section.*
☐	☒	A description of all covariates tested
☒	☐	A description of any assumptions or corrections, such as tests of normality and adjustment for multiple comparisons
☐	☒	A full description of the statistical parameters including central tendency (e.g. means) or other basic estimates (e.g. regression coefficient) AND variation (e.g. standard deviation) or associated estimates of uncertainty (e.g. confidence intervals)
☒	☐	For null hypothesis testing, the test statistic (e.g. *F*, *t*, *r*) with confidence intervals, effect sizes, degrees of freedom and *P* value noted<br>*Give P values as exact values whenever suitable.*
☐	☒	For Bayesian analysis, information on the choice of priors and Markov chain Monte Carlo settings
☒	☐	For hierarchical and complex designs, identification of the appropriate level for tests and full reporting of outcomes
☐	☒	Estimates of effect sizes (e.g. Cohen's *d*, Pearson's *r*), indicating how they were calculated

Our web collection on statistics for biologists contains articles on many of the points above.

Software and code

Policy information about availability of computer code

Data collection	No software was used in data collection.
Data analysis	All codes for data analysis, including simulation and real data analysis, are posted through GitHub at https://github.com/Jingning-Zhang/PROSPER_analysis (ref. 59). Codes, scripts, reference data, and toy example to perform PROSPER are publicly available at https://github.com/Jingning-Zhang/PROSPER (ref. 60).<br><br>The majority of our statistical analysis was performed using R 3.6.1 and R 4.0.2, and R packages: bigsnpr_1.12.2, bigstatsr_1.5.12, doMC_1.3.8, iterators_1.0.14, inline_0.3.19, RcppArmadillo_0.12.6.4.0, Rcpp_1.0.11, MASS_7.3-60, glmnet_4.1-8, Matrix_1.6-1.1, SuperLearner_2.0-28.1, gam_1.22-2, foreach_1.5.2, nnls_1.5, caret_6.0-94, lattice_0.21-8, ggplot2_3.4.3, stringr_1.5.0, readr_2.1.4, bigreadr_0.2.5, optparse_1.7.3. We used python 3.8.2 for implementing PRS-CSx. We used PLINK2 for computing PRS.

For manuscripts utilizing custom algorithms or software that are central to the research but not yet described in published literature, software must be made available to editors and reviewers. We strongly encourage code deposition in a community repository (e.g. GitHub). See the Nature Portfolio guidelines for submitting code & software for further information.

Data

Policy information about availability of data

All manuscripts must include a data availability statement. This statement should provide the following information, where applicable:

- Accession codes, unique identifiers, or web links for publicly available datasets
- A description of any restrictions on data availability
- For clinical datasets or third party data, please ensure that the statement adheres to our policy

The PRSs developed for traits in GLGC and AoU will be released through the PGS Catalog (https://www.pgscatalog.org) with publication ID PGP000595 and score IDs PGS004622-PGS004686 upon publication.

Simulated genotype data for 600K subjects from five ancestries are available at https://dataverse.harvard.edu/dataset.xhtml?persistentId=doi:10.7910/DVN/COXHAP.

GWAS summary level statistics for five ancestries from GLGC are available at http://csg.sph.umich.edu/willer/public/glgc-lipids2021/results/ancestry_specific/. GWAS summary level statistics for three ancestries from AoU are available at https://dataverse.harvard.edu/dataset.xhtml?persistentId=doi:10.7910/DVN/FAWEQK. GWAS summary statistics for the 23andMe discovery data set could be made available through 23andMe to qualified researchers under an agreement with 23andMe that protects the privacy of the 23andMe participants. Please visit https://research.23andme.com/collaborate/#dataset-access/ for more information and to apply to access the data.

GRCh37 and GRCh38 reference genome data from Phase-3 1000 Genome Project (1000G) are available at https://www.internationalgenome.org/data. Access to UKBB individual level data can be requested from https://www.ukbiobank.ac.uk/enable-your-research/apply-for-access.

Supplementary Data files and Source Data files are provided with this paper.

Human research participants

Policy information about studies involving human research participants and Sex and Gender in Research.

Reporting on sex and gender	We did not perform sex- and gender- based analysis.
Population characteristics	Participants in this study were recruited from the customer base of 23andMe, Inc. All these individuals included have provided informed consent and answered surveys online according to our human subject protocol reviewed and approved by Ethical & Independent Review Services, a private institutional review board (http://www.eandireview.com). The analyses contain GWAS data of five ancestries, genetically predicted by pipeline from 23andMe, Inc., including European, African American, Latino, East Asian, and South Asian. The detailed sample size for each ancestry used for training, tuning and validation is provided in Supplementary Data 7-8.<br><br>We use the individuals with available quality control and sample relateness information in All of Us to generate GWAS summary statistics, which is then used as training of PRS. The detailed sample size for each ancestry is provided in Supplementary Data 7.<br><br>For the analysis using training data from GLGC and AoU, we use individuals from UK Biobank for tuning and validation purpose of the PRS. The detailed sample size for each ancestry is provided in Supplementary Data 8.
Recruitment	The individuals included in our analyses are part of the 23andMe, Inc. participant cohort. All these individuals included have provided informed consent and answered surveys online according to our human subject protocol reviewed and approved by Ethical & Independent Review Services, a private institutional review board (http://www.eandireview.com).<br><br>GWAS summary statistics from the Global Lipids Genetics Consortium are downloaded from http://csg.sph.umich.edu/willer/public/glgc-lipids2021/results/ancestry_specific/. All of Us data set is accessed through All of Us Research Program (https://allofus.nih.gov/). The UK Biobank data is obtained under the UK Biobank resource application 17712.
Ethics oversight	All individuals from 23andMe included have provided informed consent and answered surveys online according to our human subject protocol reviewed and approved by Ethical & Independent Review Services, a private institutional review board (http://www.eandireview.com).<br><br>All participants from UK Biobank provided written informed consent (more information is available at https://www.ukbiobank.ac.uk/2018/02/gdpr/).<br><br>The information of individuals from All of US included in our analyses has been collected according to All of Us Research Program Operational Protocol (https://allofus.nih.gov/sites/default/files/aou_operational_protocol_v1.7_mar_2018.pdf). Detailed consent process of All of Us is described on https://allofus.nih.gov/about/protocol/all-us-consent-process.<br><br>The data from GLGC is publicly available GWAS summary statistics without individual-level information.

Note that full information on the approval of the study protocol must also be provided in the manuscript.

Field-specific reporting

Please select the one below that is the best fit for your research. If you are not sure, read the appropriate sections before making your selection.

☒ Life sciences ☐ Behavioural & social sciences ☐ Ecological, evolutionary & environmental sciences

For a reference copy of the document with all sections, see nature.com/documents/nr-reporting-summary-flat.pdf

Life sciences study design

All studies must disclose on these points even when the disclosure is negative.

Sample size

We conducted a simulation study for the ideal sample size of individual level datasets, and results are shown in Supplementary Figure 13. Generally, the sample size of individual level datasets for tuning purpose within the range of 1000-3000 is adequate. We used individual level data from 23andMe and UKB, and their sample sizes meet this requirement. Detailed sample sizes are listed below.

We analyzed existing data from four main sources: 23andMe, GLGC, AoU, and UKBB.
The averaged sample size from 23andMe for five ancestry populations are EUR (N =2700K), African American (N =136K), Latino (N =442K), East Asian (N =116K), and South Asian (N =31K). The ratio of sample sizes for training, tuning and validation is roughly about 7:2:1, and detailed numbers are in Supplementary Data 7-8.
The averaged sample size from GLGC for five ancestry populations are EUR (N =931K), AFR (N =93K), EAS (N =146K), and SAS (N=34K). Detailed numbers are in Supplementary Data 7.
The averaged sample size from AoU for three ancestry populations are EUR (N =48K), AFR (N =22K), and Latino/Admixed American (N=15K). Detailed numbers are in Supplementary Data 7.
The averaged sample size from UKBB for five ancestry populations are EUR(N=20K), AFR (N=8K), EAS (N=2K), SAS (N=10K), and AMR (N<1K). Detailed numbers are in Supplementary Data 8. Due to small sample size of AMR, we excluded it from the reported results.

Data exclusions

The analyses were restricted to unrelated individuals. Individuals were defined as related if they shared more than 700 cM identity-by-descent (IBD), including regions where the two individuals share either one or both genomic segments IBD. This level of relatedness (roughly 20% of the genome) corresponds approximately to the minimal expected sharing between first cousins in an outbred population. More details of selecting unrelated individuals within 23andMe data is provided in the Supplementary Note in a previous manuscript available at https://www.biorxiv.org/content/10.1101/2022.03.24.485519v5.abstract.

The quality control of All of Us data is conducted by All of Us research team. Detailed information about genotyping, ancestry determination, quality control, removing related individuals are listed in All of Us Research Program Genomic Research Data Quality Report (https://support.researchallofus.org/hc/en-us/articles/4617899955092-All-of-Us-Genomic-Quality-Report-).

For tuning and testing purpose of PRS using individuals in UKBB, we exclude participants with missing phenotype data for each trait/disease in its corresponding analysis.

Replication

We've ensured reproducibility of our analysis findings by thoroughly documenting our methods and data processing. The code for data analyses is available at https://github.com/Jingning-Zhang/PROSPER_analysis. The package of PROSPER is available at https://github.com/Jingning-Zhang/PROSPER.

The simulation studies were repeated 3 times for all candidate methods, and all of them produced similar outcomes.

The trait of height is analyzed in both 23andMe and All of Us (AoU) datasets by 2 times. The performances of the proposed PROSPER method outperforms all other alternatives in both data analyses, and all other methods also have similar performance in the two data analyses.

In addition, we avoid overfitting and enhance replicability of by using independent validation samples randomly selected from the corresponding populations. For example, 23andMe data are randomly split into independent training and validation datasets The model is trained using the GWAS summary level statistics from training dataset; then tested the independent validation dataset. For the analyses with the GLGC and AoU, the model is trained using the their GWAS summary statistics; then tested in a independent UK Biobank dataset.

Randomization

Not applicable. This is an observational study

Blinding

Not applicable. This is an observational study.

Reporting for specific materials, systems and methods

We require information from authors about some types of materials, experimental systems and methods used in many studies. Here, indicate whether each material, system or method listed is relevant to your study. If you are not sure if a list item applies to your research, read the appropriate section before selecting a response.

Materials & experimental systems

n/a	Involved in the study
☒	☐ Antibodies
☒	☐ Eukaryotic cell lines
☒	☐ Palaeontology and archaeology
☒	☐ Animals and other organisms
☒	☐ Clinical data
☒	☐ Dual use research of concern

Methods

n/a	Involved in the study
☒	☐ ChIP-seq
☒	☐ Flow cytometry
☒	☐ MRI-based neuroimaging
